# Enhancing Accuracy in Distinguishing Bipolar and Unipolar Disorders: Using MDQ Subscales and BSDS

**DOI:** 10.1192/j.eurpsy.2025.1061

**Published:** 2025-08-26

**Authors:** K. Kim, H. Lim, E. Moon

**Affiliations:** 1 Department of Psychiatry and Biomedical Research Institute, Pusan National University Hospital, Busan; 2 Department of Psychiatry, Pusan National University School of Medicine, Yangsan; 3 Department of Psychiatry and Biomedical Research Institute, Pusan National University Hospital, Pusan, Korea, Republic Of

## Abstract

**Introduction:**

Distinguishing between bipolar and unipolar disorder is essential for effective treatment, yet accurate diagnosis remains challenging despite extensive research. The MDQ (Mood Disorder Questionnaire) and BSDS (Bipolar Spectrum Diagnostic Scale) are widely used self-assessment tools, each offering unique advantages. However, these tools are typically used based on total scores, potentially overlooking valuable information within individual items.

**Objectives:**

This study aims to employ clustering analysis on the MDQ and BSDS, utilizing subscales derived from factor analysis, to better differentiate patients with bipolar and unipolar disorders.

**Methods:**

The study included patients diagnosed with bipolar and bipolar depression, with diagnoses confirmed by a psychiatrist according to DSM-IV-TR criteria. A total of 299 patients with bipolar depression and 142 with unipolar depression completed the MDQ and BSDS. Based on prior factor analysis, the MDQ was divided into two subscales: the positive activation subscale (items 3, 4, 8, 9) and the negative activation subscale (items 1, 2, 6, 7, 12, 13). K-means clustering was performed twice: once using the total scores from the MDQ and BSDS (two scores), and using the positive activation subscale, negative activation subscale from the MDQ, and the total score from the BSDS (three scores). The analysis was iterated 1000 times to avoid overfitting.

**Results:**

The analysis identified an optimal solution with K=2. Cluster 1, characterized by high scores on both questionnaires, predominantly comprised bipolar patients. In contrast, Cluster 2, with lower scores, was primarily composed of unipolar patients. Using the total scores from both the MDQ and BSDS for clustering yielded an accuracy of 67.88%. In the second analysis using the MDQ subscales and the BSDS total score, the accuracy improved to 77.55%.

**Conclusions:**

Clustering based on the MDQ and BSDS achieved a 77.55% accuracy in distinguishing bipolarity when using MDQ subscales alongside the BSDS score, demonstrating a promising level of precision with self-report questionnaires. Importantly, segmenting the MDQ into positive and negative activation subscales resulted in a nearly 10% increase in accuracy compared to using total scores alone. This suggests that increasing the dimensionality of the data by incorporating disorder-specific subscales can improve clustering accuracy. These findings highlight the potential of using high-dimensional psychiatric data to develop more effective classification models.

**Disclosure of Interest:**

None Declared

